# Mr. Ambrose Richard Trevor McEnnery

**Published:** 1911-05-13

**Authors:** 


### Obituary.

Mr. Ambrose Richard Trevor McEnnery, surgeon, Royal Mail Steam Packet Company, has died in London. Mr. McEnnery took his L.S.A. in 1902 and the L.M.S.S.A. in 1907. He studied at Bristol and St. Mary's, and had been both senior surgeon and assistant medical examiner to the above-named Company.

